# Parental imprisonment, childhood behavioral problems, and adolescent and young adult cardiometabolic risk: results from a prospective Australian birth cohort study

**DOI:** 10.1186/s40352-025-00329-5

**Published:** 2025-04-30

**Authors:** Michael E. Roettger, Jolene Tan, Brian Houle, Jake M. Najman, Tara McGee

**Affiliations:** 1 School of Demography, The Australian National University, 146 Ellery Crescent, Acton, ACT 2601 Australia; 2 MRC/Wits Rural Public Health and Health Transitions Research Unit (Agincourt), School of Public Health, Faculty of Health Sciences, University of the Witwatersrand, Johannesburg, South Africa; 3 School of Public Health, Public Health Building, The University of Queensland, Herston, 4006 Australia; 4 School of Criminology and Criminal Justice, Griffith University, 176 Messines Ridge Road, Mount Gravatt, QLD 4122 Australia

**Keywords:** Parental imprisonment, Behavioral problems, Body mass index, Systolic blood pressure, Diastolic blood pressure, Waist circumference, Cardiometabolic risk

## Abstract

**Objectives:**

Recent studies have demonstrated that parental imprisonment (PI) is associated with cardiometabolic risk later in life. However, underlying risk factors for these associations have not previously been explored. Using a life course framework, the present study explores how early childhood emotional and behavioral dysregulation and PI may be associated with progressive cardiometabolic risk factors in adolescence and young adulthood among male and female respondents in an Australian birth cohort.

**Methods:**

The study follows a subset of 7,223 live, singleton births from 1981 to 1984 in Brisbane, Australia where data was collected on parental imprisonment at ages 5 & 14 and behaviors from the Child Behavioral Checklist (CBCL) at age 5. Our sample examines 1,884 males and 1,758 females whose mothers completed prenatal, age 5, and age 14 interviews and respondents completed one or more interviews at a health clinic at ages 14, 21, and 30. Multivariate regression was used to examine cross-sectional results, while individual growth models examined longitudinal patterns.

**Results:**

Dividing the analysis by sex, we examined how parental imprisonment was potentially mediated or moderated by CBCL subscale measures for aggression, social-attention-thought (SAT) disorders, internalizing, and depression. No associations were found among male respondents. Among female respondents, controlling for these behaviors, there was a significant association between parental imprisonment and higher systolic blood pressure at age 30, while all CBCL measures were found to moderate waist circumference at age 30 and BMI at ages 14, 21, and/or 30. Using individual growth curve modelling, we observed the increased CBCL aggression and SAT scores were increasingly associated with higher BMI as respondents aged in adulthood.

**Conclusions:**

Using prospective cohort data, our results suggest that PI and emotional and behavioral dysregulation are associated with BMI, systolic blood pressure, and waist circumference in females, along with potentially increasing levels of cardiometabolic risk, as measured by increased BMI, from age 14 through age 30. The result is suggestive of the importance of examining early emotional/behavioral problems and PI as joint risk factors for developing cardiometabolic risk factors that may progress into cardiometabolic diseases at later stages in the life course.

## Introduction

Parental imprisonment (PI) is associated with a range of adverse behavioral and health outcomes across the life course. Studies have shown that PI is associated with childhood aggression and internalizing behaviors, along with increased risk for mental health problems and antisocial and delinquent behaviors in adolescence and adulthood (Boch & Ford, 2018; Metcalfe et al., 2023; Murray & Farrington, 2008; Shehadeh et al., 2016; Swisher & Roettger, 2012; Wakefield & Wildeman, 2011). Physical health outcomes, including asthma, childhood sleep problems, increased BMI and blood pressure, sedentary behaviors, sexually transmitted infections, physical disability, and premature mortality, are also linked with having a parent imprisoned (Jackson & Vaughn, 2017; Le et al., 2019; Lee et al., 2013; Roettger & Boardman, 2012; Roettger et al., 2022; van de Weijer et al., 2018, 2021; Wildeman et al., 2018). PI is also associated with a complex array of adversities and outcomes, such as poorer academic performance, social exclusion, and criminal justice involvement that compound over the life course, leading to cumulative disadvantages that may impact health and well-being (Foster & Hagan, 2013; Giordano, 2010; Roettger & Dennison, 2018). Depending on the level of adversity experienced by children, PI may either directly lead to poorer health outcomes, or act as an indicator for poorer health (Jackson et al., 2021). While most studies have utilized cross-sectional methodologies, a few studies have examined how parental imprisonment may be linked with risk factors for the development of non-communicable cardiometabolic diseases in young adulthood or middle-aged populations (Roettger & Boardman, 2012; Roettger et al., 2022, 2023; Tung et al., 2023). One recent longitudinal study examining PI and obesity through age 15 among a poor urban U.S. sample found an association between PI and reduced childhood obesity risk among African Americans, but not white or Hispanic children (Li & Colen, 2024); however the study did not examine associations into adulthood. Ongoing research into PI and potential linkages to cardiometabolic diseases like diabetes, stroke, and heart failure is critical to inform for treatment and intervention strategies leading to long-term health of children who have experienced parental incarceration.

The prevalence of imprisonment in the United States (U.S.), the United Kingdom (U.K.) and Australia has prompted calls to focus on PI as both a potential cause of health disparities and as a public health issue for chronic health conditions, with a need for further research (Beresford et al., 2020; Wildeman & Andersen, 2017). In Australia, an estimated 4% of all children and up to 20% of Indigenous children may experience PI by age 16 (Dennison et al., 2013). In the U.S., one-third of young adults aged 18–29 reported experiencing parental incarceration, with 4% of white and 25% of African American children having experienced a parent who spent one or more years in prison (Enns et al., 2019; Sykes & Pettit, 2014). In England and Wales, an estimated 310,000 children (~ 2.5% of all children under age 17) per year experience PI (Kincaid et al., 2019).

The overwhelming majority of studies on the health outcomes of parental incarceration are from U.S.-based samples, where adult imprisonment rates are approximately four times higher than other Western countries such as Australia, New Zealand, Canada and the U.K. (Leigh, 2020a; Wildeman et al., 2018). The current study, situated in Australia, has an imprisonment rate similar to other non-US Western countries, but has experienced a doubling in imprisonment rates from 100 persons per 100,000 adults in the 1980s to 220 per 100,000 adults in 2020 (Leigh, 2020b). Currently, convicted Australian prisoners serve an average of 3.7 years in prison (Leigh, 2020b). Indigenous Australians are incarcerated at rates similar to those of African Americans in the U.S., although the size of the Indigenous population, at 3% of the total population, makes examining parental imprisonment in the Indigenous subpopulation untenable in general Australian population surveys (Leigh, 2020a). International studies on parental imprisonment and long-term child outcomes are uncommon, and can assist with expanding results of U.S. studies to examine the applicability of patterns and theories outside of U.S. populations in a broader context of Western countries (Besemer et al., 2019).

It is important to acknowledge that those who experience parental incarceration also experience a range of other related risk factors. Research on adverse childhood experiences (ACEs; of which PI is one) provides some insight regarding how PI may be linked with cardiovascular and metabolic diseases. A life course perspective suggests that early exposure to the stress of PI, in combination with other ACEs, may lead to cardiometabolic diseases in later life (Ben-Shlomo & Kuh, 2002; Kivimäki et al., 2023). Research examining this link demonstrates that *stress*, along with genetic and lifestyle factors, increases the risk of later cardiovascular disease (Deschênes et al., 2021; Suglia et al., 2020). A recent national study of American adolescents found that 51% of adolescents who experienced PI (vs. 14% of adolescents not experiencing PI) reported having four or more ACEs, a common marker for identifying those at heightened cardiovascular risk (Ryan et al., 2023). Additionally, a recent literature review found that adults reporting four or more ACEs were at twice the risk for experiencing cardiovascular disease and premature death (Godoy et al., 2021). The link between ACEs and cardiometabolic diseases have been found to be mediated by risk factors that include low SES, externalizing behaviors, substance use, poor diet and lifestyle factors, and biological strain and premature ageing, all of which have been found to occur more frequently in children experiencing PI (Del Toro et al., 2022; Del Toro et al., in press; Foster & Hagan, 2007; Heard-Garris et al., 2018; Jackson et al., 2022; Jackson & Vaughn, 2017; Järvinen et al., 2024; Niño & Cai, 2020; Roettger et al., 2011; Wildeman, 2010). Recent studies have also found that depression and anxiety mediate the association between ACEs (including PI) and cardiovascular disease (Bertele et al., 2022; Deschênes et al., 2021), with PI associated with increased risk of anxiety and depression in the existing literature (Gifford et al., 2019; Lee et al., 2013). This points to the importance of examining behavioral and emotional dysregulation as a possible moderator of the relationship between PI and cardiometabolic indicators; the focus of the current study. By identifying the links between PI and risk factors over time, it is possible to understand how PI and related stressors may lead to cardiometabolic disease as individuals progress into later stages of the life course. Behavioral and emotional dysregulation in those who experience PI is more likely due to the stressors of PI and other ACEs, leading to development of biological strain in later life (Johnson & Arditti, 2023).

Biological measures of stress associated with PI and cardiometabolic risk at various stages in the life course include early puberty, reduced telomere length, high levels of C-Reactive Protein and higher allostatic load (Boch & Ford, 2014; Del Toro et al., 2022; Niño & Cai, 2020; Tither & Ellis, 2008; Tung et al., 2023). Within this context, theories of stress and adversity associated with parental imprisonment can help to explain various aspects of how PI may be linked with development of cardiometabolic risk. Foster and Hagan have emphasized that PI incorporates a range of stressors at the individual, family, school, and community levels that may lead to multi-dimensional risk factors that progress over time (Foster & Hagan, 2015, 2017). Giordano and Copp (2015) have labeled these composite risk factors as “packages of risk,” where PI compounds individual and family adversities, such as parental absence, family violence, residential instability, parental mental illness, parental substance abuse, child mental illness, child delinquency, and childhood poverty (Arditti, 2015; Giordano, 2010; Giordano & Copp, 2015; Tasca et al., 2011; Wildeman et al., 2012), along with institutional and neighborhood factors, such as living in a food desert, neighborhood economic deprivation from high rates of imprisonment, or lacking a quality educational environment at school (Wilson, 2009). While the “package of risk” postulate has been principally used to explain how combinations of social risk factors, not just PI, may lead to poorer adult outcomes, the idea can be extended to include biological risk mechanisms and outcomes. The accumulation of stress over the life course, through a combination of “packages of risk” that include factors such as social strain, social exclusion, exposure to offending, and low SES, have been observed to exacerbate ageing and increase earlier risk for cardiometabolic diseases (Boen, 2020; Boen et al., 2023; Yang et al., 2016). Some limited research on parental imprisonment has found that parental imprisonment and health outcomes are moderated by a range of factors, such as other ACEs and substance use in adolescence (Jackson et al., 2021; Roettger & Houle, 2021). Thus, adopting a multi-dimensional, cumulative stress framework to examine potential pathways from parental imprisonment to cardiometabolic risk will advance life course research in this area.

Given that the effect of stressors on biology may vary by sex, the link between PI and cardiometabolic risk may also be differentiated by sex. Research from several data sources on parental and familial incarceration find that that cardiometabolic risk markers and diseases are concentrated in females (Boch & Ford, 2014; Connors et al., 2020; Lee et al., 2014; Roettger & Boardman, 2012; Roettger et al., 2022, 2023), with only one study linking familial incarceration with ischemic heart disease in males at mid-life (White et al., 2016). High levels of C-Reactive protein, a measure of biological stress and cardiometabolic disease risk, is associated with parental imprisonment for female children, but not males, which suggests these associations may vary by sex (Boch & Ford, 2014). Given this we would expect that there may be an association between social stressors and cardiometabolic risk for females but not males. Results from studies examining sex variations for PI find that stress-related cardiometabolic risk factors, including BMI, waist circumference, blood pressure, and C-Reactive Protein are concentrated in adolescent and young adult females; these results potentially indicate that biological stress-markers associated with PI manifest as increased risk for early cardiovascular and metabolic diseases in females who have experienced PI, when compared to females who have not experienced PI (Boch & Ford, 2018; Roettger & Boardman, 2012; Roettger et al., 2022; Tither & Ellis, 2008). We note that several studies examining PI and cardiometabolic risk have utilized pooled-sex data, while failing to test variations by sex (Branigan & Wildeman, 2019; Järvinen et al., 2024; Lee et al., 2013; Li & Colen, 2024; Tang et al., 2023; Tung et al., 2023). These studies may yield inaccurate results or fail to detect underlying sex-based cardiometabolic risk if the link between PI and cardiometabolic risk is found only among females. Efforts to test for sex variations between PI and cardiometabolic disease fit broader trends within medicine and epidemiology, where there is an effort to examine cardiometabolic and other non-communicable disease by sex as more recent analyses have shown existing pooled-sex or male-only studies suppressing knowledge of sex-specific pathways that lead to diabetes mellitus, obesity-related cancers, and heart failure in females (Carcel et al., 2024). Analyzing sex-specific patterns for PI and cardiometabolic disease are thus situated in broader epidemiological and medical research identifying sex-based pathways to cardiometabolic diseases.

From a life course perspective, a multi-dimensional, cumulative stress framework can help to understand how PI and emotional and behavioral dysregulation may lead to cardiometabolic risk in females. As we discuss above, evidence suggests that cardiometabolic risk may develop from behavioral and psychiatric disorders associated with obesity and BMI gain (Gonzalez et al., 2012; Korczak et al., 2013; Mamun et al., 2009), while other research suggests that ACEs are associated with a range of childhood emotional and behavioral problems (Greeson et al., 2014; Kerker et al., 2015). Additionally, while longitudinal research suggests that delinquency and depression, measures of internalizing and externalizing behaviors, differentiate risk for increased BMI among females who have first experienced PI as adolescents or adults (Roettger & Boardman, 2012; Roettger et al., 2023); the potential interrelationships between PI and early childhood emotional and behavioral dysregulation have, to our knowledge, not been explored. Notably, some research has linked childhood internalizing and externalizing behaviors, measures of emotional and behavioral dysregulation, with the emergence of cardiometabolic risk in adolescent girls (Louise et al., 2012a, b). Therefore we hypothesize that PI and cardiometabolic risk for females, in adolescence and adulthood, may be moderated by high levels of emotional and behavioral problems in childhood.

**Fig. 1:**
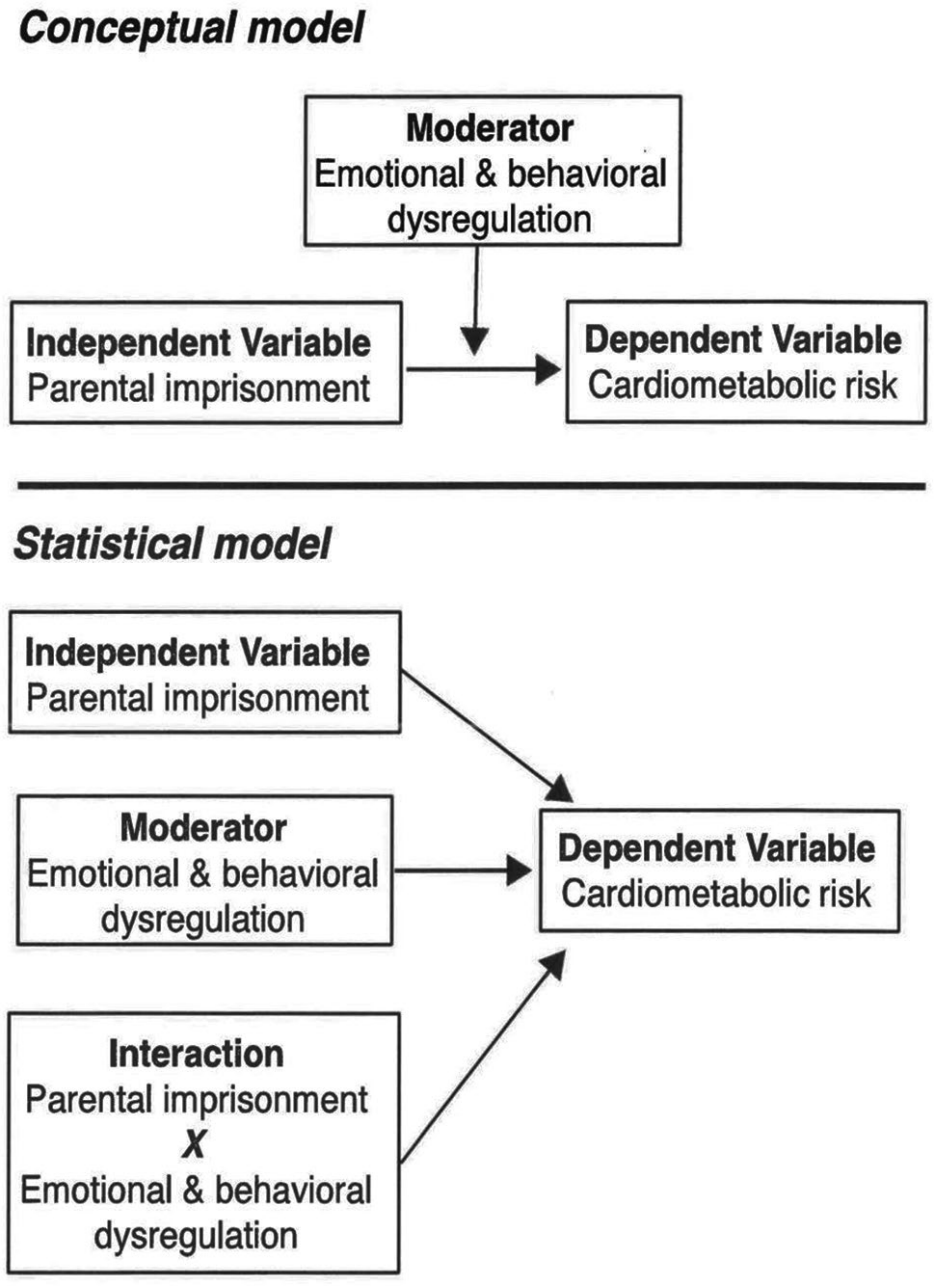
Conceptual and statistical models testing emotional and behavioral dysregulation as a moderator of parental imprisonment in the prediction of cardiometabolic risk

As Johnson and Arditti (2023) have noted, parental imprisonment is a “complex process” which involves a range of factors and issues, described above as “packages of risk,” that cumulatively lead to stress over the life course. The focus of the current study, as shown in Fig. 1, explores the direct effect of PI on cardiometabolic risk indicators and the moderating effect of emotional and behavioral dysregulation on this relationship through the inclusion of interaction terms in the moderation model. As outlined in the research literature above, both PI and emotional and behavioral dysregulation are linked to cardiometabolic disease risk in later life. Using six domains of behavioral and emotional dysregulation in early childhood, we test if these and PI interact to increase cardiometabolic risk in adolescence and adulthood. As shown in our Conceptual Model in Fig. 1, this involves emotional and behavioral dysregulation as influencing the relationship between PI and cardiometabolic risk. As shown in the Statistical Model in Fig. 1, this involves adding PI, emotional and behavioral dysregulation, and an interaction of PI and emotional and behavioral dysregulation in predicting measures of cardiometabolic risk. Based on existing research on PI, we hypothesize that these factors when observed among female respondents will increase cardiometabolic risk in adolescence and early adulthood. While we are not able to fully test the underlying complex processes and “packages of risk” associated with PI and their relationship with cardiovascular disease in later adulthood, these analyses: provide the opportunity to identify potential pathways through which behavioral and emotional dysregulation may function; examine variation in risk by sex; and better understand how cumulative stress may manifest as biological health adversities later in the life course.

### Aims of the current study

The present study aims to examine if the cumulative risk of behavioral and emotional dysregulation in early childhood and exposure to parental imprisonment compound to increase risk for cardiometabolic risk. Further it aims to examine whether there are variations by biological sex. Earlier research by Roettger et al. (2022) examined the relationships between exposure to parental imprisonment from age 5 to 14 to and cardiometabolic risk using data from the Mater Hospital-University of Queensland Study of Pregnancy. Roettger et al. (2022) found a direct association between parental imprisonment at age 5 or younger and measures of cardiometabolic risk at ages 14, 21, and 30 in pooled-sex and female-only analyses, but no associations were found in the analysis of males. For parental imprisonment measured up to age 14, one association was found between PI and increased BMI for female-only analyses. Building on the research that childhood behavioral problems can predict a range of adversities in adulthood, the present study examines if the association between PI and cardiometabolic risk is moderated by childhood problem behaviors, with potential variation by respondent sex. In doing so, we examine the moderation effect of age 5 emotional and behavioral dysregulation (moderation effect) for those experiencing parental incarceration up to age 14 (independent variable), on cardiometabolic disease risk measures in adolescence and young adulthood (dependent variables). We then examine whether emotional and behavioral dysregulation combines with parental incarceration (interaction effect) to increase cardiometabolic risk. Lastly, we investigate whether cardiometabolic risk may increase over time by looking at cardiometabolic risk at different ages across the life course (dependent variables). In doing so, this study helps to understand and provide insights into how cumulative stress from PI (and unobserved related risks) and emotional and behavioral dysregulation may lead to the development of cardiometabolic risk in adolescence and adulthood.

In existing research looking at PI and cardiometabolic disease, a number of methodological issues arise such as a lack of data on the timing of parental imprisonment and related events and a lack of repeated prospective assessments of both parents and children. Many of these studies also suffer from measurement limitations (e.g., childhood traumas, emotional/behavioral issues using non-validated scales, self-reported height and weight) and have high attrition or low statistical power that impede the determination of causality (Tang et al., 2023; Thornberry, 2009, 2020; Wildeman et al., 2018). Our study progresses this research by exploring if parental imprisonment and childhood emotional and behavioral dysregulation predicts increased cardiometabolic risk in a prospective birth cohort with mother-reported measures of parental imprisonment, validated measures of child behavior, and clinically gathered biometric data. To address issues arising from potential selection bias and attrition in the study, we adopt an exploratory approach to analysis and use a set of procedures to assess the validity of the results which compare those respondents experiencing PI with those not experiencing PI.

The present research literature links PI with a range of emotional and behavioral dysregulation. While exploratory, we examine the extent to which differing types of emotional and behavioral dysregulation may predict higher cardiometabolic risk at different ages, from adolescence to young adulthood. This may help to illuminate potential pathways leading to cardiometabolic risk. To assess the effect of emotional and behavioral dysregulation, we explore if variation in aggression, internalizing, Social-Attention-Thought (SAT) disorders underlying autism, ADHD, and anxiety, and depression may differentially moderate the relationship between PI and cardiometabolic risk.

While our exploratory analysis is unable to prove causation and address the complex interplays between social/family adversity, emotional and behavioral problems, and adult outcomes, this analysis suggests potential from which future theoretical and empirical work may progress research in this area. At present, evidence links PI and cardiometabolic risk, but evidence is needed to develop a comprehensive understanding of pathways and mechanisms which may lead a large number of children experiencing PI to developing chronic health conditions in later life.

## Data and methods

We use data from the Mater Hospital-University of Queensland Study of Pregnancy (MUSP). The original study contains 7,223 children born in live, singleton births between 1981 and 1984 in Brisbane, Australia, with several subsequent waves of data collection of both mothers and children, using both respondent-completed surveys and collection of obstetric and additional biometric data collected by trained health professionals at a hospital clinic. For this study, we incorporate maternal data collected during pregnancy, when the child was age 5, and when the child was age 14, while biometric data for children were collected at ages 14, 21, and 30. At these waves of interview, biometric data was available for 3,794 child respondents at age 14, 2,336 at age 21, and 1,712 at age 30. We note that respondent inclusion for biometric data at these waves was impacted by respondents’ ability to attend the hospital clinic at the Mater Hospital for data collection due to factors such as distance, time, and access to public transport to the hospital in Brisbane. Biometric data collection at the Mater Hospital clinic was collected using standard procedures and the same machines were used to measure blood pressure and weight for all participants, reducing error that may occur with differing instrumentation, staff training, and environments (e.g., thousands of homes for in-home interviews). Further details of the MUSP data are available in the MUSP cohort profiles and research publications (Najman et al., 2005, 2014).

Following research cited above finding cardiometabolic risk primarily linked with females, but not males experiencing parental/familial incarceration, we separate analyses by biological sex at birth. To remove missing data from respondents who exit the study and have substantial not-missing-at-random data over multiple waves, our analytic sample consists of respondents whose mothers completed the prenatal interview, as well as the interviews when the child was aged 5 and 14, along with respondents having height and weight recorded at a clinic interview for at least one interview at ages 14, 21, or 30. There are 1,884 males and 1,758 females (3,652 total respondents) who meet these criteria to form the core of our analytic sample, leaving us with 1,665 males and 1,532 females (3,197 respondents) at age 14; 1,087 males and 1,117 females (2,204 respondents) at age 21; and 637 males and 844 females (1,581 respondents) at age 30. Our analytic sample thus represents 84.3%, 94.3%, and 86.5% of all respondents with biometric data, respectively, collected at ages 14, 21, and 30. Our analytic sample contains 50.1% of all respondents at birth; as a percentage of the original cohort, contains 44.0% of the original sample at age 14, 30.3% at age 21, and 21.7% at age 30. We note that those who attended the clinic for follow-up interviews at the Mater Hospital were more likely to reside near the location of their birth.

Ethics approval was received from relevant committees at The University of Queensland and the Mater Misericordiae Hospital, South Brisbane, Australia for data collection. For the present study, we use deidentified secondary data exempt from Human Ethics approval.

To maintain confidentiality, data from the MUSP are not made publicly available. Prospective users may apply for data access via the study website at: https://social-science.uq.edu.au/mater-university-queensland-study-pregnancy?p=9#9.

### Measures of cardiometabolic risk: dependent variables

#### Body mass index (BMI, kg/m^2^)

At ages 14, 21, and 30, biometric data for height (in centimeters) and weight (in kilograms) for respondents was collected by a health professional. BMI is a well-established risk factor for cardiovascular and metabolic diseases in later life, particularly when combined with other cardiovascular disease measures (Amato et al., 2013).

#### Systolic blood pressure (SBP, mmHG)

SBP at age 30 was measured during physical assessments of respondents. Two readings were taken 5 min apart when the respondents were sitting and at rest. The respondent’s SBP was the average of these two readings (Das et al., 2020). For SBP, hypertension is measured in three categories, with normal from 90 to 129 mmHG, high-normal is 130–139 mmHG, and hypertension is ≥ 140 mmHG (Conen et al., 2007).

#### Diastolic blood pressure (DBP, mmHG)

DBP at age 30 was measured during physical assessments of respondents. Two readings were taken 5 min apart when the respondents were sitting and at rest. The respondent’s DBP was the average of these two readings (Das et al., 2020). For DBP, three categories of hypertension are recorded, with normal blood pressure at ≥ 84 mmHG, high-normal is 85–89 mmHG, and hypertension is ≥ 90 mmHG (Conen et al., 2007).

#### Waist circumference (cm)

Self-reported waist circumference (centimeters) at age 30. To measure waist size, respondents were provided with a paper measuring tape and detailed instructions regarding tape placement for measuring their waist circumference. A waist circumference above 80 cm in females and 90 cm in males is associated with an increased risk of type II diabetes and cardiovascular disease (Darsini et al., 2020).

### Independent variables

#### Parental imprisonment

When the mother was interviewed when her child was aged 5 and 14, she was asked if (1) her or (2) her current resident partner (the biological father or stepfather) had ever been imprisoned. Using these four questions, we created an indicator of whether the mother or her current partner had ever been imprisoned at ages 5 and/or 14. This coding has been previously used in MUSP analysis (Roettger et al., 2022) and is used due to the low rate of imprisonment in the general population during the 1980s and 1990s when the maternal data were collected. We note that about two-thirds of children at age 5 and one-half of children at age 14 who have ever experienced parental incarceration, resided with both biological parents at the time of interview.

### Moderator variables

#### Child behavioral problems

At the interview when her child was age 5, the mother completed items from the Child Behavioral Checklist (CBCL), Ages 4–18. We use four subscales modelled from the original CBCL scales that include Aggression (10-items, α = 0.83), Internalizing (10-items, α = 0.76), Social-Attention-Thought (SAT) problems (10-items, α = 0.74), and Depression (6-items, α = 0.66). These scales have been validated in prior research and are fully described elsewhere (Najman et al., 1997; Williams et al., 1998). Among the available subscales at age 5, Aggression and Internalizing are used to capture early measures of external and internal processes of stress. The CBCL SAT problems scale measures elements of ADHD, autism, and anxiety disorders. The CBCL Depression scale is used due to its linkage as a possible modifier for how ACES are linked with cardiovascular risk in the existing literature.

### Control variables

#### Child ethnicity

Mother-reported child ethnicity, with indicators for whether the child was of Indigenous Australian or Asian descent.

#### Child sex at birth

An indicator for if the child was classified as male or female at birth.

#### Pregnancy status

Whether female offspring respondents reported being pregnant at ages 14, 21, or 30. An indicator for pregnancy is used to control for varying cardiometabolic measures which may occur in pregnancy, such as pre-eclampsia or increased BMI. Controlling for pregnancy has been done in prior research on PI and health outcomes (Roettger & Boardman, 2012; Roettger et al., 2022, 2023).

#### Mother’s education

During initial interviews, the mother reported her highest level of education. We categorized education as not completing secondary education, completing secondary education, or completing some form of post-secondary education.

#### Mother’s age at birth

Mother’s age at the birth of the study child.

#### Child birth weight (kg)

Child’s recorded weight at birth obtained from obstetric records.

#### Mother’s pre-pregnancy BMI (kg/m2)

Mother’s pre-pregnancy BMI, based on self-reported height and weight at her first interview.

### Analytic strategy

We use multivariate OLS regression to examine outcomes of (1) BMI at ages 14, 21, & 30, (2) SBP at age 30, (3) DBP at age 30, and (4) waist circumference at age 30. We test for interactions between PI and CBCL measures in a regression model, while controlling for maternal education, maternal age, maternal pre-pregnancy, respondent ethnicity, respondent pregnancy at the appropriate wave, and respondent birth weight. Due to prior studies finding PI being associated with cardiometabolic risk factors in females only, we estimate separate models for male and female respondents.

To make better use of partial data across waves and to examine if observed patterns hold over time, we also use a 2-level random effects (multi-level) model to examine potential interactions between PI and CBCL measures for BMI at ages 14, 21, and 30. The 2-level random effects model controls for repeated measures at the individual level. We also test for potential 3-way interactions for PI, age, and CBCL measures to examine if the moderating effect of CBCL behaviors becomes more prevalent in adulthood, as found in one recent paper examining, BMI, PI, and delinquency (Roettger et al., 2023).

To estimate models for multiple imputation, we use imputed chained equations (ice) in STATA to produce 75 imputed datasets, performing analysis using STATA’s ‘mi estimate’ command (Royston & White, 2011).

All analyses are conducted using STATA version 17.1.

### Attrition and assessing validity of findings

The general attrition patterns in the data for children have been reported by Najman et al. (2015, pg.78c), showing that retention drops to 40% of the original sample of children at age 30, with further attrition for biometric data. As we have outlined above, the attrition in the sample is significant over time. Prior research has found that analysis with the MUSP data at later waves yields accurate and valid results despite attrition (Najman et al., 2015). One paper, evaluating potential attrition bias in the panel, has found that attrition from the MUSP is associated with maternal education and ethnicity (Saiepour et al., 2019). Additionally, the number of respondents completing biometric data has been limited by data collection occurring at a clinic at the Mater Hospital (the hospital where children were originally born). Our study criteria for including respondents whose mothers completed interviews when the study child was aged 5 and 14 kept ~ 85–95% of total respondents for whom biometric data was collected.

While attrition and the representative population of case-controls are issues, we note that our exploratory analyses are oriented towards testing if the pattern in the data holds, compared to the group of children whose mothers did not report parental imprisonment; as Rothman and colleagues have classically noted, assessing the validity of findings for a group characteristic compared with a control lacking the group characteristic, not representativeness of a sample, is an essential component of advancing empirical knowledge in comparative research (Rothman et al., 2013). The implication is that the validity of the patterns rest on differences between the group of interest and controls, even if attrition may lead to selection bias over time. The focus of the analysis is thus on whether a group of people who have experienced PI and a control group yield valid distinctions, not whether selection bias makes the results invalid for a population. Within this context, we evaluate the validity and consistency of associations between childhood problem behaviors and parental imprisonment predicting cardiometabolic risk, using a strategy consisting of four approaches: (1) controlling for ethnicity and parental socioeconomic status, which have been found to predict attrition in the MUSP data (Saiepour et al., 2019), along with pre-pregnancy maternal BMI and child birth weight that may indicate susceptibility to cardiometabolic disease; (2) examining the extent to which the associations are consistently observed across waves and across measures of emotional and behavioral dysregulation and cardiometabolic risks, thereby seeing if patterns change over time; (3) testing if the patterns hold longitudinally for BMI at ages 14, 21, and 30 for consistency; and (4) comparing multiple imputation (MI) and complete case analysis in the longitudinal analyses for consistency. These approaches are centered on cumulatively helping to establish the validity of the findings that may be assessed through future studies.

Our fourth component for assessing validity of results is a technique meant to establish validity by comparing results from complete case analysis and from multiple imputation (MI) in our longitudinal analysis examining moderation patterns for BMI (Young & Johnson, 2015). However, caution is warranted when modeling interactions with too much missing data; by adding in random noise into interactions, multiple imputation is known to decrease the overall level of significance of moderating effects, thus potentially reducing accuracy by increasing the risk of type II error (Graham, 2012). To address missingness for respondents unable to attend the health clinic at Mater Hospital, we impute data where respondents have completed at least one clinic visit over 3 waves. As such, we add interaction terms in multiple imputation, using a “Just Another Variable” approach, to evaluate variations between complete case and MI analysis to assess the consistency of estimates (Graham, 2012). To further reduce risk of type II error from imputation of interaction models, we estimate models for cases where CBCL age 5 scores, parental imprisonment histories, and at least one non-missing BMI measure in three waves is present for respondents.

## Results

Table 1 (males) and Table 2 (females) contain the means, standard deviations, and p-values for tests used in the analysis. These analyses find that cardiometabolic risk measures do not differ by PI for males, while PI is associated with higher BMI (*p* < 0.05) and SBP (*p* < 0.05) at age 30 for females. PI is associated with higher behavioral problems in both males and females. For both males and females who have experienced PI, PI occurs more frequently among those reporting Indigenous ancestry, and is associated with lower maternal educational attainment, and a younger maternal age at birth. PI is associated with slightly lower birth weight for males, but not females in the sample.

**Table 1:** Means and standard deviations for variables used in analysis, by parental history of imprisonment in males

	Male respondents [*N* = 1,884 at birth]						
	Parental Imprisonment			No Parental Imprisonment			
	N	Mean/%	SD	N	Mean/%	SD	p-value
Dependent Variables							
Age 14							
BMI	67	20.29	4.44	1,598	20.22	3.52	0.875
Age 21							
BMI	58	24.13	4.31	1,029	24.08	4.28	0.931
Age 30							
BMI	33	27.70	5.98	604	27.19	4.91	0.561
DBP	34	75.35	8.55	591	74.85	9.42	0.762
SBP	34	131.44	11.58	591	129.75	13.03	0.461
Waist Circumference	33	94.25	14.06	590	94.22	13.09	0.990
CBCL age 5 problem behaviors							
SAT	89	5.70	3.35	1,793	5.04	3.09	0.049
Internalizing	89	4.21	3.17	1,795	3.79	2.94	0.192
Aggression	89	7.15	3.51	1,794	6.27	3.59	0.023
Depression	89	3.93	2.88	1,795	3.50	2.85	0.169
Controls							
Pregnancy age 14	89	0%		1,795	0%		
Pregnancy age 21	58	0%		1,029	0%		
Pregnancy age 30	33	0%		604	0%		
Respondent Indigenous	89	4.5%		1,747	3.8%		0.753
Respondent Asian	89	0%		1,747	4.1%		0.051
Mother tertiary education	89	21.3%		1,792	19.1%		0.597
Mother non-secondary education	89	27.0%		1,792	15.1%		0.003
Mother’s age	89	23.01	4.28	1,795	25.65	5.02	0.000
Mother’s pre-pregnancy BMI	89	22.10	3.94	1,795	21.91	3.82	0.650
Child birth weight (kg)	89	3.37	0.55	1,795	3.47	0.53	0.064

Notes: Sample size presented as the total number of respondents with non-missing data for the variable, by parental imprisonment. BMI = Body Mass Index; DBP = Diastolic Blood Pressure; SBP = Systolic Blood Pressure; CBCL = Child Behavioral Checklist; SAT = Social-Attention-Thought Disorder

**Table 2:** Means and standard deviations for variables used in analysis, by parental history of imprisonment in females

	Female respondents [*N* = 1,758 at birth]						
	Parental Imprisonment			No Parental Imprisonment			
	N	Mean/%	SD	N	Mean/%	SD	p-value
Dependent Variables							
Age 14							
BMI	66	21.61	4.62	1,466	20.94	3.90	0.171
Age 21							
BMI	48	25.04	6.45	1,069	24.14	5.28	0.249
Age 30							
BMI	39	29.15	8.77	805	26.59	6.43	0.017
DBP	37	72.45	10.34	779	68.95	9.21	0.025
SBP	37	117.14	13.63	779	110.54	12.63	0.002
Waist Circumference	37	91.34	16.87	779	86.59	15.05	0.063
CBCL age 5 problem behaviors							
SAT	75	5.52	3.24	1,678	4.36	2.95	0.001
Internalizing	76	4.33	2.85	1,682	3.65	2.91	0.045
Aggression	75	6.67	3.95	1,678	5.58	3.32	0.006
Depression	76	4.18	2.86	1,682	3.44	2.80	0.025
Controls							
Pregnancy age 14	76	0%		1,682	0%		
Pregnancy age 21	51	5.9%		1,090	3.7%		0.418
Pregnancy age 30	40	10.0%		836	11.0%		0.843
Respondent Indigenous	75	9.3%		1,643	3.9%		0.021
Respondent Asian	75	1.3%		1,643	3.35%		0.337
Mother tertiary education	76	10.5%		1,671	20.9%		0.028
Mother non-secondary education	76	21.1%		1,671	15.5%		0.194
Mother’s age	76	24.74	5.56	1,682	25.67	4.95	0.114
Mother’s pre-pregnancy BMI	76	22.19	5.58	1,682	21.87	3.84	0.482
Child birth weight (kg)	76	3.28	0.55	1,682	3.35	0.48	0.194

Notes: Sample size presented as the total number of respondents with non-missing data for the variable, by parental imprisonment. BMI = Body Mass Index; DBP = Diastolic Blood Pressure; SBP = Systolic Blood Pressure; CBCL = Child Behavioral Checklist; SAT = Social-Attention-Thought Disorder

### Cross-sectional analyses

In Table 3, where effects are reported for males, no significant main effects for PI and interactions for PI and childhood emotional and behavioral dysregulation are observed.

**Table 3:** Main and moderating effects of CBCL measures at age 5 on parental imprisonment predicting cardiometabolic disease risk in men

	BMI, Age 14		BMI, Age 21		BMI, Age 30		DBP, Age 30		SBP, Age 30		Waist Circumference, Age 30	
CBCL scales at age 5	(1)	(2)	(1)	(2)	(1)	(2)	(1)	(2)	(1)	(2)	(1)	(2)
**SAT**												
PI	-0.06	1.07	-0.17	0.31	0.27	2.18	0.43	1.55	2.23	3.90	0.03	5.41
	[-0.90, 0.77]	[-0.59, 2.74]	[-1.31, 0.96]	[-1.87, 2.49]	[-1.47, 2.00]	[-1.63, 5.99]	[-2.90, 3.75]	[-5.77, 8.87]	[-2.33, 6.80]	[-6.16, 14.0]	[-4.56, 4.63]	[-4.68, 15.5]
SAT	0.02	0.02	0.02	0.02	-0.04	-0.02	0.11	0.12	0.19	0.20	0.06	0.10
	[-0.04, 0.07]	[-0.03, 0.08]	[-0.07, 0.10]	[-0.07, 0.11]	[-0.17, 0.09]	[-0.16, 0.11]	[-0.14, 0.37]	[-0.14, 0.38]	[-0.16, 0.54]	[-0.16, 0.56]	[-0.29, 0.41]	[-0.26, 0.45]
PI x SAT		-0.20		-0.09		-0.38		-0.22		-0.32		-1.06
		[-0.46, 0.05]		[-0.41, 0.24]		[-1.04, 0.29]		[-1.48, 1.04]		[-2.05, 1.41]		[-2.83, 0.71]
**Internalizing**												
PI	-0.05	0.43	-0.16	-0.65	0.23	-0.55	0.42	-1.30	2.23	-0.96	-0.03	1.01
	[-0.89, 0.79]	[-1.02, 1.88]	[-1.29, 0.98]	[-2.43, 1.13]	[-1.50, 1.96]	[-3.53, 2.42]	[-2.90, 3.75]	[-7.09, 4.49]	[-2.34, 6.79]	[-8.91, 6.98]	[-4.61, 4.56]	[-6.89, 8.91]
Internalizing	-0.05	-0.04	-0.04	-0.05	-0.15*	-0.16*	-0.01	-0.03	-0.02	-0.06	-0.35	-0.34
	[-0.11, 0.01]	[-0.10, 0.01]	[-0.13, 0.05]	[-0.14, 0.04]	[-0.28, -0.02]	[-0.29, -0.02]	[-0.26, 0.25]	[-0.29, 0.24]	[-0.37, 0.33]	[-0.42, 0.30]	[-0.70, 0.00]	[-0.70, 0.02]
PI x Internalizing		-0.12		0.12		0.21		0.45		0.84		-0.28
		[-0.40, 0.17]		[-0.21, 0.46]		[-0.44, 0.86]		[-0.79, 1.70]		[-0.87, 2.55]		[-2.01, 1.45]
**Aggression**												
PI	-0.09	0.72	-0.19	-0.73	0.29	-0.82	0.30	0.90	2.13	-0.11	0.15	-0.24
	[-0.92, 0.75]	[-1.25, 2.69]	[-1.32, 0.94]	[-3.07, 1.61]	[-1.44, 2.03]	[-4.49, 2.84]	[-3.03, 3.63]	[-6.21, 8.00]	[-2.45, 6.71]	[-9.88, 9.65]	[-4.46, 4.75]	[-9.95, 9.47]
Aggression	0.05	0.05*	0.04	0.04	-0.03	-0.04	0.13	0.13	0.12	0.10	-0.10	-0.10
	[0.00, 0.09]	[0.00, 0.10]	[-0.03, 0.11]	[-0.04, 0.11]	[-0.14, 0.08]	[-0.15, 0.08]	[-0.09, 0.35]	[-0.09, 0.36]	[-0.18, 0.42]	[-0.21, 0.41]	[-0.40, 0.20]	[-0.41, 0.21]
PI x aggression		-0.11		0.08		0.16		-0.08		0.31		0.06
		[-0.36, 0.14]		[-0.22, 0.37]		[-0.30, 0.61]		[-0.96, 0.79]		[-0.89, 1.52]		[-1.16, 1.27]
**Depression**												
PI	-0.05	0.94	-0.16	-0.06	0.22	0.09	0.42	0.99	2.23	1.59	-0.04	2.14
	[-0.89, 0.79]	[-0.49, 2.37]	[-1.29, 0.97]	[-1.90, 1.78]	[-1.50, 1.95]	[-2.93, 3.11]	[-2.91, 3.74]	[-4.88, 6.87]	[-2.34, 6.79]	[-6.48, 9.65]	[-4.62, 4.54]	[-5.87, 10.2]
Depression	-0.04	-0.03	-0.04	-0.04	-0.16*	-0.17*	-0.05	-0.04	-0.03	-0.03	-0.37*	-0.35
	[-0.10, 0.02]	[-0.09, 0.03]	[-0.13, 0.05]	[-0.13, 0.06]	[-0.30, -0.03]	[-0.30, -0.03]	[-0.31, 0.22]	[-0.31, 0.23]	[-0.39, 0.34]	[-0.41, 0.34]	[-0.74, -0.01]	[-0.72, 0.02]
PI x depression		-0.25		-0.03		0.04		-0.17		0.18		-0.64
		[-0.55, 0.04]	0.04	[-0.42, 0.36]		[-0.69, 0.77]		[-1.56, 1.23]		[-1.73, 2.10]		[-2.58, 1.30]
Sample Size	1,619	1,619	1,058	1,058	623	623	611	611	611	611	610	610

Notes: Tables report main effect and 95% confidence intervals. All models contain controls for race/ethnicity of respondent, maternal education at birth, age of mother at birth, mother’s pre-pregnancy BMI, and respondent’s birth weight. Presented moderators interact parental imprisonment with child age 5 CBCL measures. Sample size varies by wave and cardiometabolic outcome due to missing data. All measures for child behavioral issues are based on maternal CBCL scores at age 5. PI = Parental Imprisonment; SAT = Social-Attention-Thought Disorder; CBCL = Child Behavioral Checklist

Significance levels (two-tailed): *p < 0.05 **p < 0.01 ***p < 0.001

Table 4 contains main and moderating effects for PI, childhood emotional and behavioral dysregulation, and controls for females. Results vary by age of respondent and behavioral outcome but show a fairly consistent pattern that age 5 behavioral problems moderate cardiometabolic risk. For example, with CBCL Aggression, increasing CBCL scores for females experiencing PI are associated with an increased BMI at ages 14, 21, and 30, higher SBP, and increased waist circumference (WC); with a 1-point increase in CBCL Aggression associated with an increased BMI of 0.82 kg/m^2^ (*p* < 0.001) and a WC increase of 0.34 cm (*p* < 0.05).

**Table 4:** Main and moderating effects of age 5 CBCL measures on parental imprisonment predicting cardiometabolic disease risk in females

	BMI, Age 14		BMI, Age 21		BMI, Age 30		DBP, Age 30		SBP, Age 30		Waist Circumference, Age 30	
CBCL scales at age 5	(1)	(2)	(1)	(2)	(1)	(2)	(1)	(2)	(1)	(2)	(1)	(2)
**SAT**												
PI	0.56	-0.64	0.48	-2.23	2.02	-4.25*	2.76	1.33	5.96**	9.56*	4.17	-4.66
	[-0.35, 1.46]	[-2.40, 1.12]	[-0.99, 1.95]	[-5.18, 0.71]	[-0.03, 4.06]	[-8.19, -0.31]	[-0.37, 5.89]	[-4.80, 7.45]	[1.66, 10.3]	[1.15, 18.0]	[-0.64, 8.98]	[-14.1, 4.73]
SAT	0.04	0.03	0.16**	0.13*	0.20**	0.14	0.25*	0.23*	0.14	0.17	0.40*	0.31
	[-0.02, 0.11]	[-0.03, 0.10]	[0.05, 0.26]	[0.03, 0.24]	[0.05, 0.34]	[-0.01, 0.28]	[0.02, 0.47]	[0.00, 0.46]	[-0.17, 0.44]	[-0.14, 0.49]	[0.05, 0.74]	[-0.04, 0.66]
PI x SAT		0.22		0.50*		1.18***		0.28		-0.69		1.70*
		[-0.06, 0.51]		[0.03, 0.98]		[0.55, 1.82]		[-0.74, 1.29]		[-2.08, 0.70]		[0.15, 3.25]
**Internalizing**												
PI	0.58	-1.49	0.54	-2.19	1.98	-1.52	2.85	4.48	5.69**	11.45**	3.83	-3.37
	[-0.33, 1.48]	[-3.15, 0.16]	[-0.93, 2.01]	[-4.77, 0.39]	[-0.04, 4.00]	[-4.98, 1.93]	[-0.25, 5.94]	[-0.92, 9.89]	[1.46, 9.93]	[4.05, 18.8]	[-0.91, 8.57]	[-11.6, 4.90]
Internalizing	0.03	0.01	0.11*	0.08	0.08	0.04	0.01	0.03	0.00	0.07	0.30	0.22
	[-0.04, 0.09]	[-0.06, 0.08]	[0.01, 0.22]	[-0.03, 0.19]	[-0.07, 0.24]	[-0.12, 0.20]	[-0.23, 0.25]	[-0.21, 0.27]	[-0.32, 0.33]	[-0.26, 0.40]	[-0.06, 0.67]	[-0.15, 0.59]
PI x Internalizing		0.48**		0.62*		0.91*		-0.41		-1.44		1.81*
		[0.16, 0.81]		[0.14, 1.11]		0.18	1.64	[-1.52, 0.70]		[-2.97, 0.08]		[0.10, 3.51]
**Aggression**												
PI	0.51	-1.52	0.47	-2.70	2.05*	-3.13	2.97	4.78	6.10**	11.37**	4.25	-6.16
	[-0.40, 1.41]	[-3.26, 0.23]	[-1.00, 1.94]	[-5.53, 0.14]	[0.02, 4.08]	[-6.85, 0.58]	[-0.17, 6.10]	[-1.09, 10.6]	[1.80, 10.4]	[3.34, 19.4]	[-0.54, 9.04]	[-15.1, 2.77]
Aggression	0.09**	0.07*	0.12*	0.09	0.24***	0.18**	0.03	0.05	-0.03	0.03	0.44**	0.33*
	[0.04, 0.15]	[0.02, 0.13]	[0.03, 0.21]	[-0.01, 0.18]	[0.11, 0.37]	[0.05, 0.31]	[-0.17, 0.23]	[-0.16, 0.26]	[-0.30, 0.25]	[-0.26, 0.31]	[0.14, 0.75]	[0.02, 0.65]
PI x aggression		0.31**		0.46*		0.82**		-0.29		-0.85		1.68**
		[0.08, 0.54]	0.54	[0.11, 0.81]		[0.33, 1.32]		[-1.09, 0.51]		[-1.94, 0.24]		[0.46, 2.89]
**Depression**												
PI	0.57	-1.07	0.51	-2.00	1.97	-0.27	2.88	4.40	5.74**	11.81**	3.83	-0.27
	[-0.33, 1.47]	[-2.64, 0.50]	[-0.96, 1.99]	[-4.43, 0.43]	[-0.05, 3.99]	[-3.69, 3.15]	[-0.22, 5.97]	[-0.86, 9.66]	[1.50, 9.98]	[4.62, 19.0]	[-0.91, 8.58]	[-8.33, 7.79]
Depression	0.04	0.03	0.14*	0.11	0.08	0.05	-0.05	-0.03	-0.08	-0.01	0.27	0.22
	[-0.02, 0.11]	[-0.04, 0.09]	[0.03, 0.25]	[-0.01, 0.22]	[-0.08, 0.24]	[-0.11, 0.21]	[-0.29, 0.19]	[-0.27, 0.21]	[-0.41, 0.24]	[-0.34, 0.33]	[-0.09, 0.64]	[-0.15, 0.60]
PI x depression		0.40*		0.60*		0.60		-0.40		-1.59*		1.08
		[0.09, 0.72]		[0.14, 1.06]		[-0.14, 1.34]		[-1.51, 0.71]		[-3.12, -0.07]		[-0.63, 2.78]
Sample Size	1,489	1,489	1,091	1,091	821	821	789	789	789	789	789	789

Notes: Tables report main effect and 95% confidence intervals. All models contain controls for race/ethnicity of respondent, maternal education at birth, age of mother at birth, mother’s pre-pregnancy BMI, respondent’s birth weight, and respondent’s pregnancy status. Presented moderators interact parental imprisonment with child age 5 CBCL measures. Sample size varies by wave and cardiometabolic outcome due to missing data. All measures for child behavioral issues are based on maternal CBCL scores at age 5. PI = Parental Imprisonment; SAT = Social-Attention-Thought Disorder; CBCL = Child Behavioral Checklist. Significance levels (two-tailed): *p < 0.05 **p < 0.01 ***p < 0.001

**Fig. 2:**
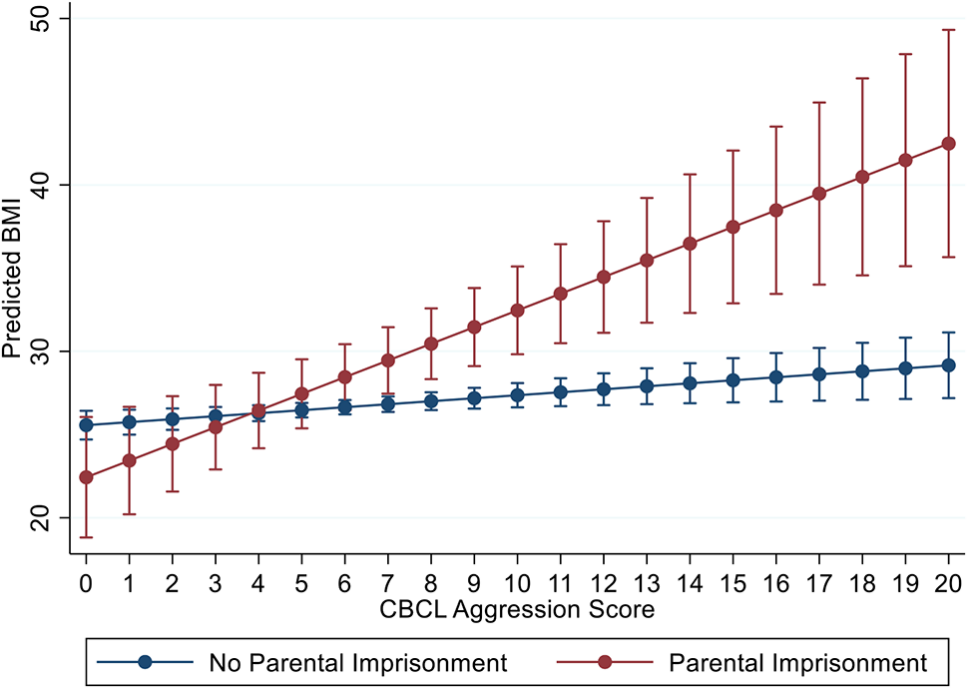
Associations between CBCL Aggression at age 5 and BMI at age 30, by parental imprisonment status among females

**Fig. 3:**
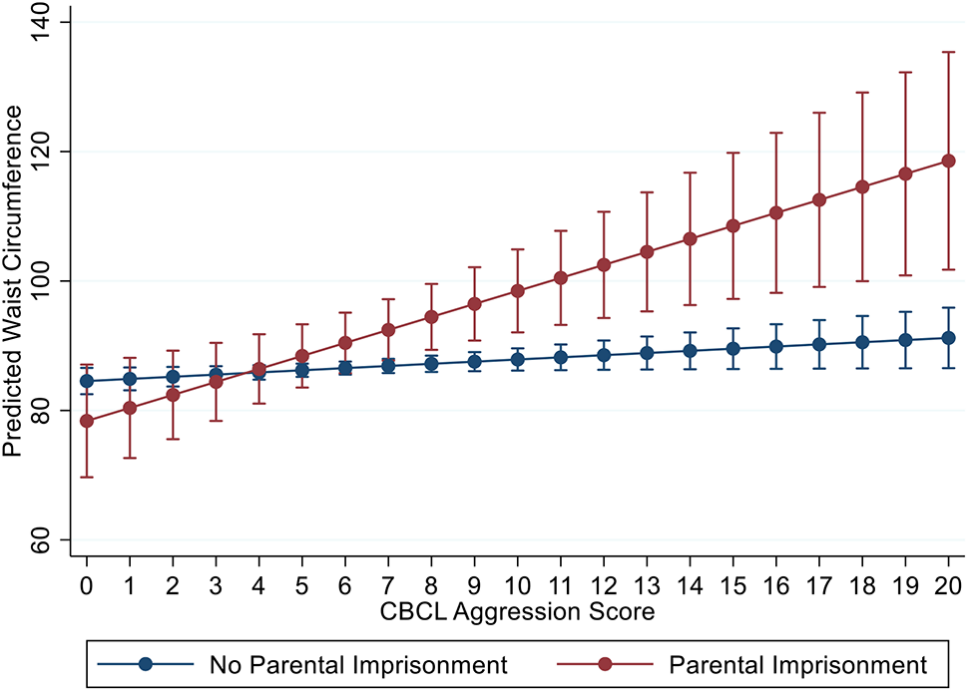
Associations between CBCL Aggression at age 5 and waist circumference at age 30, by parental imprisonment status among females

Figures 2 and 3 show the change in BMI and waist circumference associated with increasing CBCL Aggression by PI status for females at age 30. At the 0th percentile of Aggression in Fig. 2, predicted BMI is 22.43 (95% CI: 18.8, 26.05) for PI and 25.6 [95% CI: 24.7, 26.4) for those not experiencing PI; at the 90th percentile for Aggression, at a score of 11, BMI is 33.5 [95% CI: 30.5, 36.4] for PI vs. 27.5 [95% CI: 26.7, 28.4] for females not experiencing PI. For WC in Fig. 3, at the 0th percentile for CBCL Aggression, the predicted WC is 78.4 [95% CI: 69.7, 87.0] for those experiencing PI and 84.5 [95% CI: 82.5, 86.6] for those not experiencing PI; at the 90th percentile for Aggression, predicted WC is 100.5 cm [95% CI: 93.2, 107.8] for PI vs. 88.2 cm [95% CI: 86.2, 90.8] for females not experiencing PI. Both BMI and WC increase from the 0th to the 90th percentile for females irrespective of experiencing PI; however, PI at the 90th percentile for Aggression has a 5 kg/m^2^ increase for BMI and a 12.3 cm increase for WC, compared to those without histories of mother-reported PI. Examining moderation patterns for SAT, Internalizing, and Depression, where significant, yield patterns similar to those shown Figs. 2 and 3. These plots are not included due to space constraints but are available upon request.

### Longitudinal analyses

Table 5 contains the longitudinal associations between PI and BMI for female respondents. The longitudinal patterns show that Age 5 CBCL measures for SAT, Internalizing, Aggression, and Depression are significant moderators of the association between BMI and PI. The general pattern within the data is that a 1-point increase in a CBCL score is associated with an increase in BMI of ~ 0.50 kg/m^2^, with all interaction coefficients significant at *p* < 0.01 or lower.

**Table 5:** Longitudinal moderating effect of age 5 CBCL measures on parental imprisonment predicting BMI for females at ages 14, 21, and 30

CBCL scales at age 5	Main effect	Interaction effect
SAT		
PI	0.66	-1.87
	[-0.37, 1.70]	[-3.90, 0.15]
SAT	0.11**	0.09*
	[0.04, 0.19]	[0.02, 0.16]
PI x SAT		0.47**
		[0.15, 0.79]
Internalizing		
PI	0.66	-1.68
	[-0.37, 1.69]	[-3.53, 0.17]
Internalizing	0.06	0.04
	[-0.01, 0.14]	[-0.03, 0.12]
PI x Internalizing		0.55**
		[0.19, 0.92]
Aggression		
PI	0.63	-2.35*
	[-0.40, 1.66]	[-4.34, -0.35]
Aggression	0.14***	0.12***
	[0.08, 0.21]	[0.05, 0.18]
PI x aggression		0.45***
		[0.19, 0.71]
Depression		
PI	0.65	-1.27
	[-0.38, 1.68]	[-3.05, 0.52]
Depression	0.07	0.05
	[0.00, 0.15]	[-0.03, 0.13]
PI x depression		0.47*
		[0.11, 0.82]
Number of observations	3,400	3,400
Number of respondents	1,710	1,710

Notes: Tables report beta-coefficients and 95% confidence intervals. All models contain controls for race/ethnicity of respondent, maternal education at birth, age of mother at birth, mother’s pre-pregnancy BMI, respondent’s birth weight, and if the respondent was pregnant at wave of interview. The interaction terms interact parental imprisonment with child age 5 CBCL measures. All measures for child behavioral issues are based on maternal CBCL scores at age 5. PI = Parental Imprisonment; SAT = Social-Attention-Thought Disorder; CBCL = Child Behavioral Checklist

Significance levels (two-tailed): *p < 0.05 **p < 0.01 ***p < 0.001

**Fig. 4:**
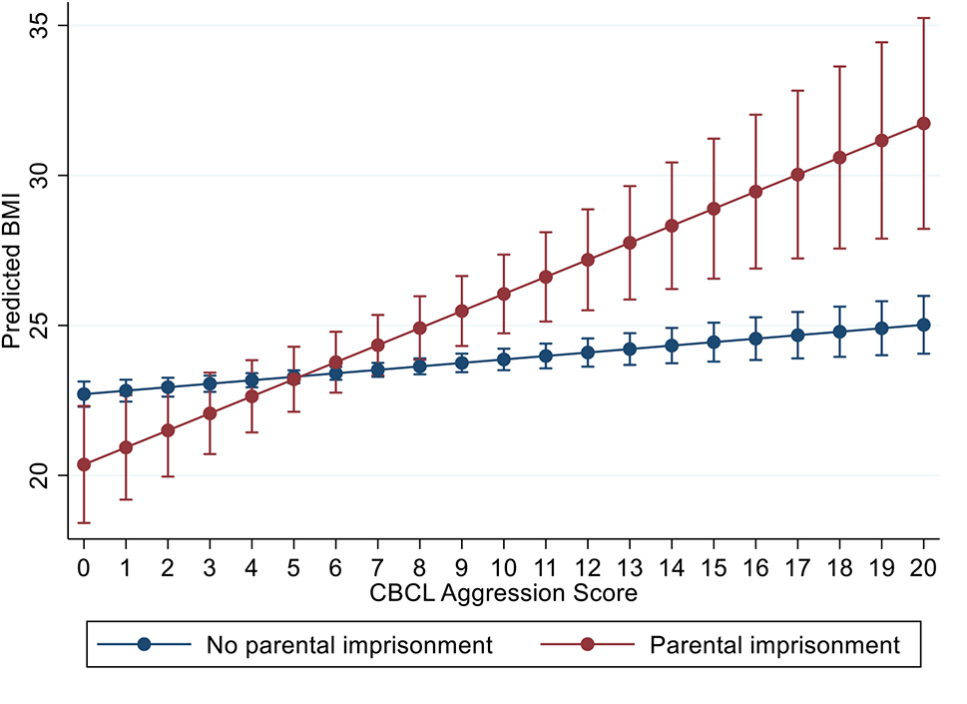
Longitudinal associations between CBCL Aggression at age 5 and BMI, by parental imprisonment status among females

Figure 4 shows the longitudinal relationship between PI and CBCL Aggression for respondents at ages 14, 21, and 30. The longitudinal association shows that PI and increasing CBCL Aggression are associated with increasing BMI. For those not experiencing PI, increasing CBCL Aggression is linked with higher BMI, though the magnitude of the association is significantly less than for those experiencing PI. The pattern is similar to the one shown in Fig. 2; similar associations linking PI and higher behavioral issues with higher BMI, are found for other reported interactions in Table 5 (for CBCL SAT, Internalizing, and Depression). These figures are available upon request.

We use multiple imputation to examine if missing data may significantly alter the patterns shown in Table 5 using complete case analysis. A comparison of the models is shown in Table 6, where data is imputed for individuals who have non-missing CBCL age 5 scores, PI histories, and had biometric data taken for at least one wave when respondents were ages 14, 21, and 30. The interactions using these imputed data increased the number of observations by 55% (3,400 to 5,277) and yielded substantially similar results to the complete case analysis.

**Fig. 5:**
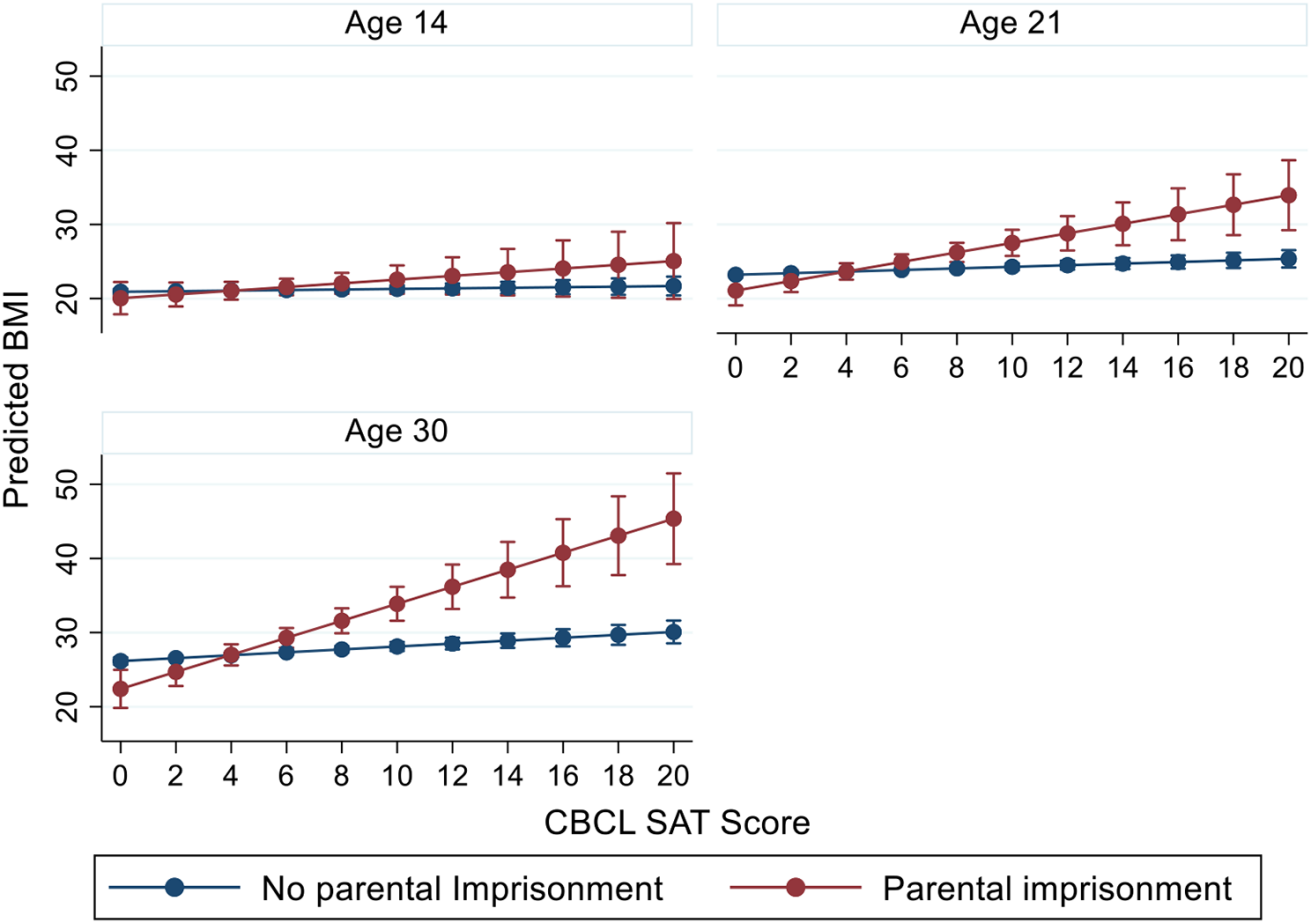
Longitudinal associations between CBCL SAT and BMI, by parental imprisonment status and age among females

One final set of analyses examined the degree to which longitudinal associations observed in Table 5 varied by age of the respondent. Adding an interaction term for age to the models that were reported in Tables 6 and 7 shows the coefficients and standard errors for significant interactions between PI, CBCL behaviors, and age. The 3-way interaction terms for SAT (*p* < 0.001) and Aggression (*p* < 0.05) show age varying effects. As shown in Fig. 5, the association between CBCL SAT problems and BMI for those experiencing PI is not significant at age 14. However, at age 21, the association between high CBCL scores and increased BMI for those experiencing PI is observed. The magnitude of this association increases again at age 30. No significant 3-way interactions for PI, age, and CBCL behaviors were observed for CBCL Internalizing and Depression.

**Table 6:** Comparison of complete case and imputed models for the longitudinal analysis

CBCL scales at age 5	Complete case	Imputed
SAT		
PI	-1.87	-1.94
	[-3.90, 0.15]	[-4.10, 0.23]
SAT	0.09*	0.11**
	[0.02, 0.16]	[0.03, 0.19]
PI x SAT	0.47**	0.44*
	[0.15, 0.79]	[0.09, 0.78]
Internalizing		
PI	-1.68	-1.47
	[-3.53, 0.17]	[-3.44, 0.50]
Internalizing	0.04	0.05
	[-0.03, 0.12]	[-0.03, 0.13]
PI x Internalizing	0.55**	0.47*
	[0.19, 0.92]	[0.08, 0.86]
Aggression		
PI	-2.35*	-2.41
	[-4.34, -0.35]	[-4.54, -0.29]
Aggression	0.12***	0.13***
	[0.05, 0.18]	[0.06, 0.20]
PI x aggression	0.45***	0.43**
	[0.19, 0.71]	[0.15, 0.71]
Depression		
PI	-1.27	-1.05
	[-3.05, 0.52]	[-2.98, 0.87]
Depression	0.05	0.06
	[-0.03, 0.13]	[-0.02, 0.14]
PI x depression	0.47*	0.38*
	[0.11, 0.82]	[-0.01, 0.77]
Number of observations	3,400	5,274
Number of respondents	1,710	1,758

Notes: Tables report beta-coefficients and 95% confidence intervals. All models contain controls for race/ethnicity of respondent, maternal education at birth, age of mother at birth, mother’s pre-pregnancy BMI, respondent’s birth weight, and if the respondent was pregnant at wave of interview. The interaction terms interact parental imprisonment with child age 5 CBCL measures. PI = parental imprisonment; SAT = Social-Attention-Thought disorders

All measures for child behavioral issues are based on maternal CBCL scores at age 5. PI = Parental Imprisonment; SAT = Social-Attention-Thought Disorder; CBCL = Child Behavioral Checklist

Significance levels (two-tailed): *p < 0.05 **p < 0.01 ***p < 0.001

**Table 7:** Longitudinal moderating effect of age 5 CBCL measures on parental imprisonment predicting BMI for females by respondent age

CBCL scales at age 5	SAT	Aggression
Interaction Coefficients		
PI	1.66	-0.96
	[-2.06, 5.38]	[-4.58, 2.66]
CBCL Behavior	-0.10	-0.07
	[-0.24, 0.04]	[-0.19, 0.05]
Age	0.33***	0.32***
	[0.30, 0.36]	[0.28, 0.35]
PI x CBCL Behavior	-0.44	0.03
	[-1.03, 0.16]	[-0.45, 0.50]
PI x Age	-0.18*	-0.07
	[-0.34, -0.02]	[-0.22, 0.09]
CBCL Behavior X Age	0.01***	0.01***
	[0.00, 0.02]	[0.00, 0.01]
PI x CBCL Behavior x Age	0.05***	0.02*
	[0.02, 0.07]	[0.00, 0.04]
Number of observations	3,402	3,402
Number of respondents	1,710	1,710

Notes: Tables report main effect and 95% confidence intervals. All models contain controls for race/ethnicity of respondent, maternal education at birth, age of mother at birth, mother’s pre-pregnancy BMI, respondent’s birth weight, and if the respondent was pregnant at wave of interview. The interaction terms interact parental imprisonment with child age 5 CBCL measures. All measures for child behavioral issues are based on maternal CBCL scores at age 5. PI = Parental Imprisonment; SAT = Social-Attention-Thought Disorder; CBCL = Child Behavioral Checklist. Significance levels (two-tailed): *p < 0.05 **p < 0.01 ***p < 0.001

## Discussion

In this study, we have used a prospective Australian cohort study to examine the association between PI, early childhood emotional and behavioral dysregulation, and cardiometabolic risk in adolescence and young adulthood. In our cross-sectional analysis, we found that controlling for early childhood emotional and behavioral dysregulation leads to an association between PI and SBP, while high levels of childhood problem behaviors moderate the association between PI and increased risk of BMI at ages 14, 21, and 30 and waist circumference at age 30. The moderating effect of early childhood emotional and behavioral dysregulation is observed in longitudinal analyses for BMI, showing that increasing scores for CBCL SAT, Aggression, Depression, and Internalizing scores at age 5 are associated with higher BMIs for females experiencing PI; results from MI yield substantively similar outcomes to complete case analysis, suggesting robustness of the association. For CBCL SAT and Aggression, these longitudinal associations emerge in early adulthood and increase in magnitude by age 30. For SAT, the longitudinal pattern is consistent with the cross-sectional findings where the associations become significant at age 21 and increase at age 30. These patterns are observed for female respondents, but no associations between PI and early childhood behaviors on cardiometabolic risk measures are observed among male respondents through age 30.

These patterns help to advance existing research in several significant ways. The MUSP provides a unique opportunity to prospectively combine both maternal reports of imprisonment and early childhood behaviors with later biomarkers of cardiovascular and metabolic risk in adolescence and adulthood in a birth cohort, bringing together separate bodies of research linking PI with childhood emotional and behavioral dysregulation and progressive cardiometabolic risk in females as they age. This suggests a potential pathway through which PI and high levels of childhood behavioral problems may lead to cardiometabolic disease over the life course. (Murray et al., 2014; Roettger & Boardman, 2012; Roettger et al., 2022). The use of longitudinal modeling and comparison with MI data improves upon cross-sectional studies, a major limitation of existing studies examining PI and health outcomes (Wildeman et al., 2018; Wildeman & Lee, 2021). The consistency and significance (*p* < 0.01) of a 1-point increase in a CBCL problem behavior score with a ~ 0.50 kg/m^2^ increase in BMI at ages 14, 21, and 30 suggests that high levels of a range of emotional and behavioral dysregulation, including CBCL SAT, Aggression, Internalizing, and Depression, represent a potentially substantive cardiometabolic disease risk pathway in females experiencing PI during adolescence and adulthood. The findings that an increased BMI is associated with PI in females emerges in early adulthood and continues to develop is observed in one other study using U.S. data by Roettger et al. (2023) is notable given the general pattern of increasing BMI levels in the general population during the second and third decades of life (Yang et al., 2021). Our findings suggest that cardiometabolic disease is a potentially hidden health risk in childhood which may be observed decades later in the life course after PI is experienced, above-and-beyond the normal weight gain experienced among those who do not experience PI.

Taking into account the potential multilevel, complex nature of cumulative stressors on cardiometabolic risk that accumulate over the life course associated with PI, we are able to frame our findings in a manner that may benefit future policy and interventions. Interventions to treat childhood emotional and behavioral dysregulation may improve health, but long term outcomes across the life course from established interventions are generally unknown (McClelland et al., 2017). As PI is classified as both an ACE and a component of the stress process leading to early biological aging; ameliorating these stressors may help to reduce cardiometabolic risk in later life (Austin et al., 2022; Foster & Hagan, 2013; Niño & Cai, 2020). Parental imprisonment may be both a direct risk factor for cardiometabolic disease and part of a “package of risks” that lead to cardiometabolic diseases in later adulthood, thus potentially following the associations between 4 + ACES and cardiometabolic diseases in later life (Giordano & Copp, 2015; Godoy et al., 2021; Jackson et al., 2021; Turney, 2014). Interventions and policies which target psychosocial and ecological risks linked to PI and cardiometabolic diseases, including poor diet, sedentary behaviors, education, access to health care and learning healthy behaviors, and treating substance use disorders, may reduce cardiometabolic and other health risks (Beresford et al., 2020; Foster & Hagan, 2017; Heard-Garris et al., 2018; Jackson & Vaughn, 2017; Turney, 2015).

Understanding how PI may differentially impact health by sex is critical for understanding how progression of cardiometabolic risk disease unfold over time. This research is part of a broader focus on understanding how sex differences are central to progressing research on non-communicable disease in health and medicine, generally (Carcel et al., 2024; Mauvais-Jarvis et al., 2020). The findings by sex are consistent across multiple studies and suggest that PI is associated with observed cardiovascular risk in females, but not males. Some pooled findings have linked parental imprisonment to elevated C-Reactive Protein, blood pressure and BMI, but these pooled effects have been shown to be attributed to heightened risk in females, but not males in analyses where males and females are analyzed separately (Boch & Ford, 2018; Roettger & Boardman, 2012; Roettger et al., 2022, 2023; Tung et al., 2023). While direct studies have not identified the cause for these variations, evidence for sex-based variations in behavioral stress response, where males are more likely to externalize stress and females are more likely to internalize behaviors is suggestive (Roettger & Boardman, 2012; Roettger et al., 2022; Smith et al., 2018). Males who experience parental imprisonment are more likely to be delinquent, engage in substance use, and be involved in the criminal justice system, while females are more likely to have higher rates of mental illness (Roettger et al., 2011; Swisher & Roettger, 2012). In one recent study, females who experienced parental imprisonment and were delinquent also had outcomes similar to males, while, in contrast, females who experienced parental imprisonment and were not delinquent had a higher BMI (Roettger et al., 2023). Compared to males, cardiovascular risk is delayed prior to menopause, with females more likely to experience hypertension and obesity as potential pathways to cardiometabolic diseases (Connelly et al., 2021; Davis et al., 2023). This suggests that parental imprisonment may increase cardiometabolic diseases via risk factors for increased BMI and blood pressure, both of which are observed in the current study.

For practitioners, the associations between PI, childhood emotional and behavioral dysregulation, and the emergence of cardiometabolic risk in adolescence and adulthood among females provides an opportunity to potentially reduce cardiometabolic disease risk. Research reviews of PI increasingly note the complexity of factors that impact children and the cumulative impact of these factors on child development over various stages of the life course (Giordano & Copp, 2015; Johnson & Arditti, 2023; Roettger & Dennison, 2018). Potential interventions may include therapeutically addressing emotional and behavioral problems observed in early childhood; the development of skills for self-care through diet, exercise, and healthy behaviors; and health screening for psychiatric problems and biomarkers of stress such as the C-Reactive Protein. Addressing the “packages of risk” linked with PI may also be beneficial. This may include addressing adverse childhood experiences, familial and economic stresses, biological strain, academic problems, and other issues, via the adoption of wraparound services that holistically meet the needs of children (Axelson et al., 2020). Policies and practices that reduce social exclusion associated with PI may also be beneficial (Besemer & Dennison, 2019). In later adolescence and adulthood, screening females with a family history of PI for emotional and behavioral dysregulation and cardiometabolic risk may create opportunities for interventions that reduce risk factors like obesity and blood pressure for cardiometabolic diseases like diabetes and heart failure later in the life course.

### Limitations and future directions

We note that these results need to be considered in the context of several limitations. The attrition in the study and relatively small number of respondents in the sample with histories of parental imprisonment may lead to these findings being a data artifact. The study lacks specific timing data for parental imprisonment and childhood behavioral problems, so we were unable to examine if timing of parental imprisonment and childhood behavioral problems is causally linked with cardiometabolic risk. Our study identifies individuals based on biological sex at birth and not gender identity which may lead to differences in risk. Due to small numbers of Indigenous and Asian subpopulations in the MUSP data, the study is unable to investigate if these patterns may vary by ethnicity. This study focuses on examining the potential patterns between childhood emotional and behavioral dysregulation, parental imprisonment, and subsequent cardiovascular risk, foregoing evaluation of the role of risk and protective factors, such as economic deprivation or close familial ties that might influence these risks. There remains the potential for selection bias, as parental imprisonment often occurs alongside health risks in the “packages of risk” such as parental separation, economic deprivation, and food insecurity. Future studies with more comprehensive measures on additional adversities could extend this work by examining “packages of risk” that develop later in the life course such as substance use, housing insecurity, and adult imprisonment to better understand these complex, interrelated mechanisms. Due to a relatively small sample size for PI in the MUSP data, we are unable to explore, in detail, how a broader set of risk factors at multiple levels may lead to cumulative stress that progresses into increasing cardiometabolic risk over the life-course. Lastly, this study is unable to follow individuals into later periods of the life course when cardiovascular and metabolic diseases may be directly observed; the addition of retrospective or administrative data on parental imprisonment in cohort studies at later stages in the life course may help to illuminate this issue.

Future research is needed in this area to confirm the overall general pattern of findings, including examining outcomes by racial and ethnic status, cross-national research to validate findings, and greater understanding of the complex, multilevel stress mechanisms which may lead to increased cardiometabolic risk over time. The lack of prospective cohort data with measures of childhood behavior and adult biometric data is currently a major limitation for research on parental imprisonment and child health. Potential way to address this limitation include the addition of administrative data on parental imprisonment, the collection of biometric data in social science surveys, and the extension of cohort studies. While research has linked parental imprisonment with early mortality and disability (van de Weijer et al., 2018, 2021), examining death records for cause of death and health records in older populations would help to link parental imprisonment to the burden of cardiovascular and metabolic diseases in later life. Current studies on parent and familial imprisonment have generally found that cardiometabolic disease and risk is found primarily in females, but it remains unclear if increased risk for cardiometabolic diseases associated with parental imprisonment emerge for males in later life; however, the strong link between PI and child imprisonment, along with an increase in cardiovascular diseases and imprisonment in later life are suggestive that PI is potentially associated with cardiometabolic disease in men (Coleman et al., 2021; Rosen et al., 2008; Ting et al., 2022). Lastly, examining factors leading to resiliency to cardiometabolic diseases among those who have experienced PI may aid in promoting resilience and altering health trajectories for those with cardiometabolic risk.

## Conclusion

Using a prospective birth cohort, the present study finds that early childhood emotional and behavioral dysregulation moderates the association between PI and SBP and moderates the associations between PI and BMI and waist circumference among female respondents in cross-sectional analyses. These moderation patterns hold in time-varying models for BMI, with social-attention-thought and aggressive behavioral problems found to emerge in young adulthood for female respondents at ages 21 and 30. No associations are found for male respondents. These findings suggest that policies and interventions targeting childhood behavioral problems, along with related stressors at the individual, family, and institutional/community levels, may help to mitigate cardiometabolic diseases as females progress through the life course. Future research is needed to identify potential underlying causal mechanisms and pathways, examining how parental imprisonment and childhood behavioral problems may lead to progressive cardiometabolic disease risk over the life course by age, sex, and the social determinants of health.

## Data Availability

Data from the Mater Hospital-University of Queensland Study of Pregnancy (MUSP) are not publicly freely available due to privacy and ethical issues. Researchers wishing to access the MUSP data may apply for data access via the study website maintained by the University of Queensland at: https://social-science.uq.edu.au/mater-university-queensland-study-pregnancy?p=9#9.

## Abbreviations

ACE: Adverse Childhood Experience
BMI: Body Mass Index
CBCL: Child behavioral checklist
cm: centimeter
DBP: Diastolic Blood Pressure
kg: kilogram
m: meter
MI: multiple imputation
mmHG: millimeters of mercury
PI: parental imprisonment
SAT: Social-Attention-Thought
SBP: Systolic Blood Pressure
